# Aerobic and anaerobic cellulase production by *Cellulomonas uda*

**DOI:** 10.1007/s00203-016-1230-8

**Published:** 2016-05-06

**Authors:** Henrik Vestergaard Poulsen, Fillip Wolfgang Willink, Kjeld Ingvorsen

**Affiliations:** Department of Bioscience, Section for Microbiology, Aarhus University, Ny Munkegade 116, 8000 Aarhus C, Denmark

**Keywords:** Cellulose degradation, Cellulase, *Cellulomonas uda*, Facultative anaerobe, Aerobic/anaerobic

## Abstract

*Cellulomonas uda* (DSM 20108/ATCC 21399) is one of the few described cellulolytic facultative anaerobes. Based on these characteristics, we initiated a physiological study of *C. uda* with the aim to exploit it for cellulase production in simple bioreactors with no or sporadic aeration. Growth, cellulase activity and fermentation product formation were evaluated in different media under both aerobic and anaerobic conditions and in experiments where *C. uda* was exposed to alternating aerobic/anaerobic growth conditions. Here we show that *C. uda* behaves as a true facultative anaerobe when cultivated on soluble substrates such as glucose and cellobiose, but for reasons unknown cellulase activity is only induced under aerobic conditions on insoluble cellulosic substrates and not under anaerobic conditions. These findings enhance knowledge on the limited number of described facultative cellulolytic anaerobes, and in addition it greatly limits the utility of *C. uda* as an ‘easy to handle’ cellulase producer with low aeration demands.

## Introduction

Cellulose, a linear polymer of glucose molecules joined by β-1,4-glycosidic-linkages, is the most abundant biopolymer on the planet and strongly resistant to biodegradation (OSullivan 1997). Over the past few decades, the conversion of cellulose-containing biomass to various fuel products has been actively researched as a sustainable solution to future energy demands (Himmel et al. 2010). However, the efficient use of cellulosic materials requires a costly enzymatic pretreatment, presenting a major obstacle to economical production of cellulose-based biofuels (Wilson 2009). The vast majority of described cellulase-producing microorganisms are either aerobes or anaerobes, and members of these two groups produce cellulase by very different strategies. The aerobes generally excrete their cellulolytic enzymes, whereas most of the anaerobes possess cell-bound organelle-like structures (cellulosomes), containing several catalytic units on a common scaffold (Himmel et al. 2010).

The only cellulolytic facultative anaerobes reported to date are members of the genus *Cellulomonas* (Christopherson et al. 2013; Lynd et al. 2002; Stackebrandt 2006) and a few other taxa, including *Actinotalea fermentans*, formerly *Cellulomonas fermentans* (Yi et al. 2007) and *Caulobacter* sp. FMC1 (Song et al. 2013). Although *Cellulomonas* sp. are known to use a variety of soluble substrates anaerobically, as well as to degrade cellulose aerobically (Clemmer and Tseng 1986; Dermoun and Belaich 1985; Haggett et al. 1979; Marschoun et al. 1987), anaerobic growth of these organisms on cellulosic compounds has been little investigated (Dermoun and Belaich 1988; Reguera and Leschine 2001).

The genus *Cellulomonas* comprises high-GC gram-positive bacteria belonging to the phylum *Actinobacteria*. *Cellulomonas* harbours microorganisms, which produce cellulolytic enzymes, and their main habitat appears to be soil (Stackebrandt et al. 2006). *Cellulomonas uda* has been reported to grow and produce cellulases under both aerobic and anaerobic conditions. This was our rationale for choosing this strain as a potential microorganism for low-cost production of cellulase enzymes in simple fermenters with limited stirring and aeration capacity. The present study investigates the growth and cellulase production of *C. uda* under aerobic and anaerobic growth conditions during cultivation on α-cellulose.

## Materials and methods

### Strains and growth medium

The strains used were *C. uda* (DSM 20108) purchased from the German Collection of Microorganisms and Cell Cultures (DSMZ, Braunschweig, Germany), and *Cellulomonas* sp. ATCC 21399 (DSM 20108) obtained from the American Type Culture Collection (Manassas, Virginia, USA). These two strains are identical and should both be referred to as *C. uda*.

The basal growth medium (referred to as BM) contained (per litre): NaCl, 1.5 g; (NH_3_)_2_SO_4_, 6.2 g; (Na)_2_HPO_4_, 9.1 g; KH_2_PO_4_, 0.9 g; EDTA, 50 mg; MgSO_4_·7H_2_O, 0.2 g; ZnSO_4_·7H_2_O, 8 mg; FeSO_4_·7H_2_O, 20 mg; MnSO_4_·H_2_O, 15 mg; CaCl_2_·2H_2_O, 26 mg; MOPS, 41.8 g; and yeast extract, 300 mg. Prior to autoclaving (121 °C for 20 min), the pH was adjusted to 7.4 with 5 M NaOH. Biotin (1 mg l^−1^) and thiamine (1 mg l^−1^) were aseptically added to the autoclaved medium from filter-sterilized stock solutions. Cellulosic growth substrates, α-cellulose (Sigma, C-8002) and Avicel (Merck, Microcrystalline, 1.02331.0500), were added prior to autoclaving, whereas the soluble growth substrates, glucose and cellobiose, were added aseptically to the autoclaved medium through a 0.22-µm filter.

### Cultivation conditions

All cultivations were performed in 500-ml Erlenmeyer flasks fitted with a glass tube sidearm sealed by a butyl rubber stopper with an aluminium seal (Bellco Glass Inc., Vineland, New Jersey, USA), through which samples were aseptically drawn by a syringe. The initial culture volume was 100 ml, and unless stated otherwise, the inoculum was a 5-ml sample of a culture grown to stationary phase on α-cellulose (10 g l^−1^) in the same medium. Aerobic incubations were performed in flasks fitted with paper stoppers (Steristopfen^®^, Heinz Herenz, Hamburg, Germany); in the anaerobic incubations, these were replaced by thick butyl rubber stoppers and the headspace was thoroughly flushed with pure nitrogen gas through a sterile filter (0.22 µm). All cultures were incubated at 30 °C in a rotary shaker at 120 rpm at high air humidity—accordingly loss of growth medium due to evaporation was minimal during aerobic cultivations (approx. 0.1 ml day^−1^).

Prior to sampling, the incubation flasks were left unshaken for 2 min to settle most of the particulate cellulosic substrate. The samples were centrifuged for 5 min at 11,300×*g,* and the supernatant was removed for later measurements of extra-cellular protein, ammonium concentration and cellulase activity. For quantification of carbon and nitrogen, pellets from representative samples (no settling of particulate material) were repeatedly washed in phosphate buffer (0.1 mol l^−1^, pH 7.0). Unless immediately analysed, samples were freeze-stored at −20 °C.

### Quantification of extra-cellular protein

Concentrations of extra-cellular protein in culture supernatants were determined by the microbiuret method described by Goa (1953), using bovine serum albumin as a standard.

### α-Cellulose utilisation in *C. uda* cultures

To assess the degree of α-cellulose utilisation (after 240 h incubation), the C/N ratio of the solids was analysed as follows: cells and remaining cellulose were pelleted by centrifugation (11,300×*g* for 5 min), washed 3 times in phosphate buffer (0.1 mol l^−1^, pH 7.0) and finally resuspended in a known volume of MilliQ water. Representative subsamples (125 µl) were transferred to tin capsules, dried and analysed for C and N content using a CN analyser (vario EL cube, Elementar, Hanau, Germany). From the N concentration, the C contribution from cells to the total C content was approximated using a C/N ratio of 5.3 determined for cellobiose-grown *C. uda* (aerobically) using the same C/N analyser. The C content of the residual cellulose was then estimated as the difference between the total C content of the solids and the C contributed by the cells.

### Cell dry weight (CDW) calculated from C/N analysis or ammonium assimilation

The CDW was calculated in one of two ways: (1) C/N analysis of the solids or (2) ammonium assimilation.

1. The C/N analysis was performed as described in the above subsection, ‘*α*-*Cellulose utilisation in C. uda cultures*’. In samples from cultures grown on soluble substrates (glucose and cellobiose), the CDW concentration was directly calculated from the measured C-concentration in the solids and subsequently converted to CDW by assuming 0.5 g C g^−1^ CDW (Stanbury et al. 1995). In samples from cultures containing α-cellulose, the amount of C in cell biomass was first calculated from the measured N concentration and the C/N ratio of *C. uda* cells (experimentally determined as 5.3 by the method described in the previous subsection) and then converted to CDW concentration by assuming 0.5 g C g^−1^ CDW.
2. The amount of N assimilated for cell growth was calculated by subtracting the N content of extra-cellular protein (assuming 0.16 g N g^−1^ protein) (Stoppok et al. 1982) from the total amount of N assimilated as ammonium. The CDW was then calculated as described above, using the cellular C/N ratio of 5.3.

### Quantification of fermentation products

The concentrations of the fermentation products were determined in appropriately diluted samples of culture supernatant using a HPLC Dionex Ultimate 3000 system (Thermo Scientific, Waltham, Massachusetts, USA), equipped with an Aminex HPX-87H column operated at 50 °C. The eluent was 5.0 mmol l^−1^ sulphuric acid administered at 0.6 ml min^−1^. The organic acids were detected by the system’s UV detector, and ethanol, glucose and cellobiose were detected by an external refractive index detector (RI-101 Shodex, New York, USA).

### Filter paper activity (FPA)

The FPA was measured by a modification of Ghose’s (1987) procedure: 1.0 ml of phosphate buffer (0.1 mol l^−1^, pH 7.0) was added to 0.5 ml of culture supernatant in an Eppendorf tube and preheated to 40 °C. After inserting a strip of Whatman No. 1 filter paper (1 cm × 6 cm, ~50 mg), the tubes were incubated at 40 °C for 2 h. Each analysis was accompanied by enzyme and substrate blanks (devoid of culture supernatant and paper strip, respectively) as controls. The amount of reducing sugar released from the paper strip was determined by a modification of the 3.5-dinitrosalicylic acid (DNS) method (Miller 1959). One FPA unit is the amount of enzyme releasing 1 µmol glucose equivalents per minute of reaction. In all experiments, less than the critical amount of reducing sugar (2.0 mg) was released from the strip (Ghose 1987). The lower limit of reducing sugar quantified by our DNS protocol was ~70 µg ml^−1^, corresponding to a lower detection limit of ~10 U/l in the FPA assay.

### Avicelase activity (AVA)

The AVA was measured by a modification of the procedure of Ghose (1987): 1.0 ml of phosphate buffer (0.1 mol l^−1^, pH 7.0) was added to 20 mg of Avicel (Fluka, PH 101) in an Eppendorf tube and preheated to 40 °C. After addition of 0.5 ml of preheated culture supernatant, the tubes were incubated at 40 °C for 2 h. Each analysis was accompanied by enzyme and substrate blanks (devoid of culture supernatant and Avicel, respectively) as controls. The amount of reducing sugar released from the Avicel was determined by a modification of the 3.5-dinitrosalicylic acid (DNS) method (Miller 1959).

### Measurement of endoglucanase activity (EGA)

EGA (endo-1,4-β-d-glucanase) was determined using Cellazyme C tablets purchased from Megazyme (Bray, Ireland). Unless otherwise stated, the manufacturer’s instructions were followed with some modifications: 20 µl of culture supernatant (11,300×*g* for 5 min) was added to 1.0 ml of preheated phosphate buffer (0.1 mol l^−1^, pH 7.0) and incubated for 20 min at 40 °C. The reaction was stopped by adding 1.0 ml of 96 % ethanol. Enzyme blanks devoid of supernatant were included for all runs. This assay was very reproducible and exhibited a linear response over a broad range of activities and could be used for measuring EGA in both aerobic and anaerobic cultures without diluting samples. The measured activities were therefore plotted directly as OD_590_. Endoglucanase activity in aerobic cultures was measured a few times using a carboxymethylcellulose (CMC) assay as described by Ghose (1987). This method was not sufficiently sensitive to monitor EGA in anaerobic cultures.

### Ammonium concentration

The ammonium concentration of the culture supernatants was determined by the salicylate-hypochlorite method (Bower and Holm-Hansen 1980) in appropriately diluted samples.

### Reproducibility

We observed a high degree of consistency both between replicate cultures, replicate measurements and different growth experiments, and therefore, results are presented as average values. All cultivations were performed in duplicate, unless stated otherwise. The FPA, ammonium and extra-cellular protein results are reported as the average of two determinations. Measurements of the EGA and fermentation products, as well as the C/N analyses, were performed once only. The exception was the C/N analysis of α-cellulose degradation, which was performed in triplicate.

## Results

### Aerobic growth on α-cellulose

**Fig. 1:**
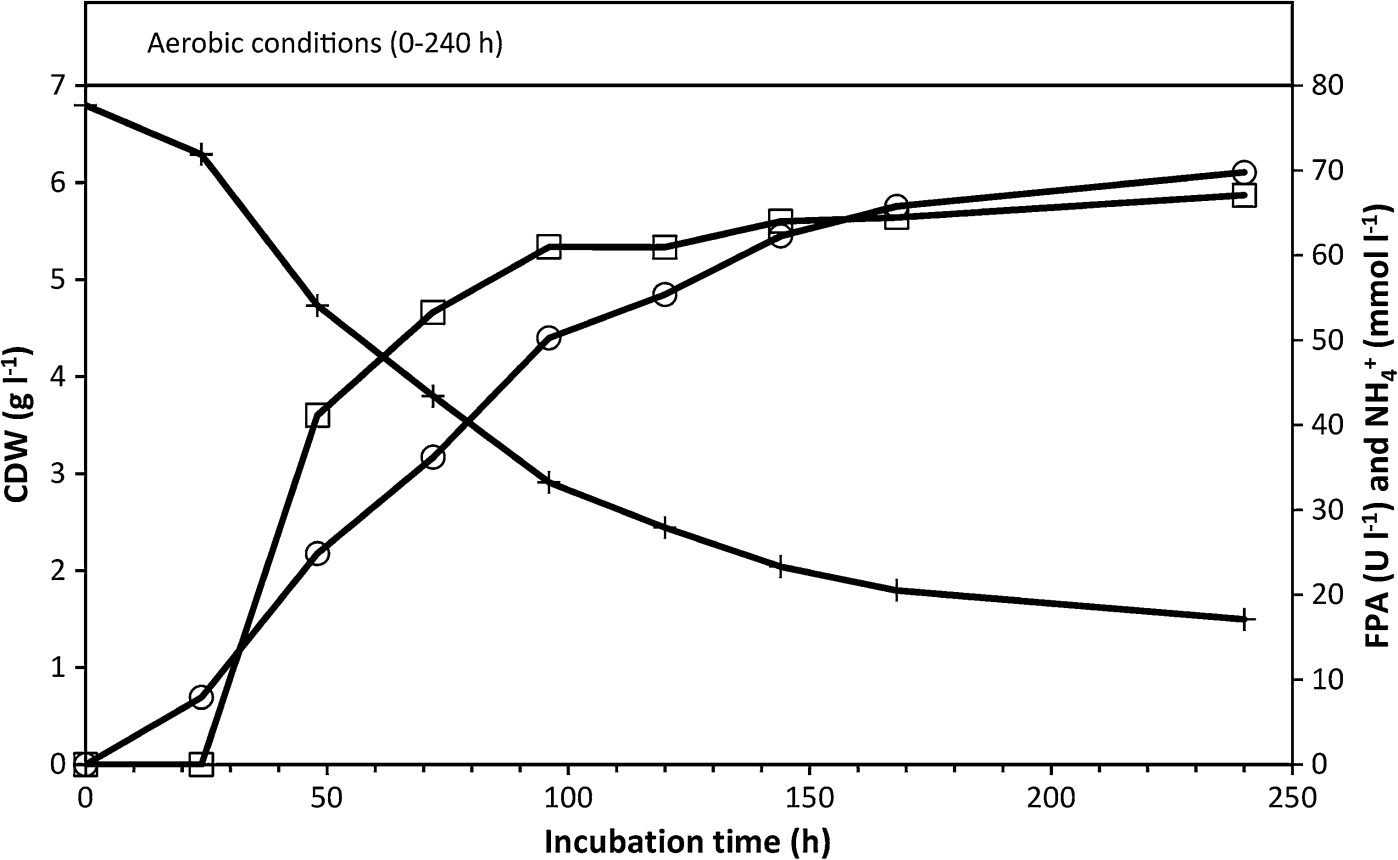
Aerobic growth of *C. uda* on α-cellulose (20 g l^−1^). Plotted are the FPA, CDW (calculated from the assimilated ammonium) and ammonium concentrations in the cultures. FPA levels below the detection limit are shown as zero. Symbols: (*open* *circle*) CDW, (*open* *square*) FPA and (*plus sign*) NH_4_^+^

**Fig. 2:**
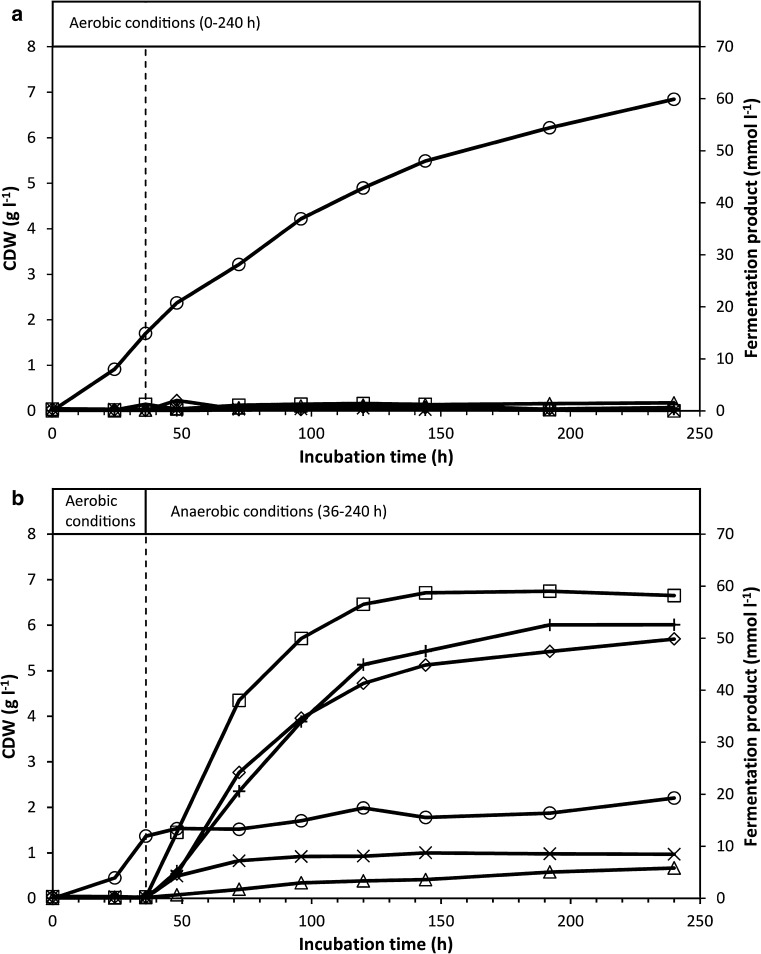
Concentrations of fermentation products and CDW in cultures of *C. uda* grown on α-cellulose (20 g l^−1^) under aerobic conditions (**a**) or under aerobic conditions for the first 36 h, followed by anaerobic conditions (**b**). The *broken vertical line* in (**b)** indicates the switch to anaerobic conditions. The experiment is the same as in Fig. 3. Symbols: (*open* *square*) Formic acid, (*open* *diamond*) Acetic acid, (*open* *triangle*) Lactic acid, (*multiple sign*) Succinic acid, (*plus sign*) Ethanol and (*open* *circle*) CDW

In aerobic incubations grown on α-cellulose (20 g l^−1^), the FPA sharply increased after approximately 24 h, levelling off at ~60 U l^−1^ after approximately 100 h, followed by a slight increase throughout the remaining incubation period (Fig. 1). During the incubation period, the pH decreased from 7.4 to 6.8 (further decreases were prevented by the MOPS buffer; in the absence of MOPS, the pH would have declined to ~5.2 after 48 h of aerobic incubation with 20 g l^−1^ α-cellulose). No acidic metabolites were produced during aerobic growth (see Fig. 2a); therefore, the pH decline was attributed to assimilation of ammonium ions for biosynthesis. Following the sampling at 240 h, glucose (10 g l^−1^) was aseptically added to the cultures. The ensuing rapid glucose consumption further reduced the pH from 6.8 to approximately 6.3 at 264 h, and ammonium assimilation resumed (results not shown).

Under aerobic conditions, 77 and 81 % of the α-cellulose initially provided at 10 and 20 g l^−1^, respectively, were degraded over 10 days. Based on these results, the growth efficiencies (g CDW g^−1^ substrate consumed) of aerobic cultures with 10 and 20 g l^−1^ α-cellulose were calculated as 0.42 and 0.41, respectively. Considering the high amounts of extra-cellular protein produced, the substrate incorporation efficiencies (g cell produced material g^−1^ substrate consumed) were estimated at 0.53 and 0.49, respectively.

### Growth and cellulase production under a shift from aerobic to anaerobic conditions

**Fig. 3:**
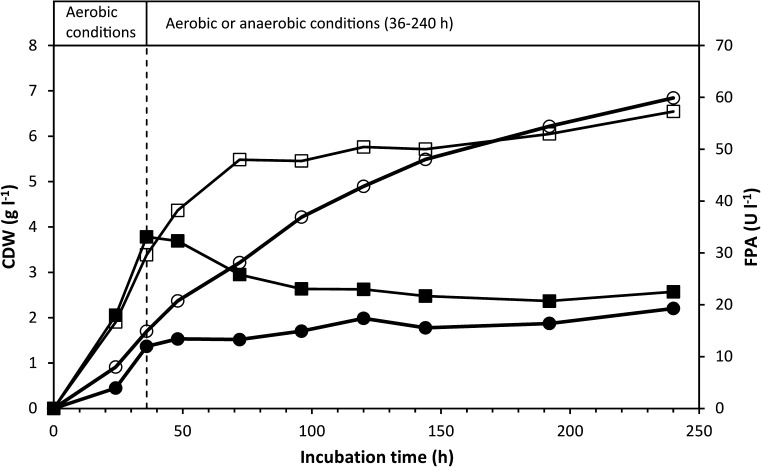
CDW (calculated from ammonium assimilation) and FPA levels in cultures of *C. uda* grown on α-cellulose (20 g l^−1^) under aerobic conditions (‘Aerobic’) or grown under aerobic conditions for the first 36 h, followed by anaerobic conditions (‘Anaerobic’). The *broken vertical line* indicates the switch to anaerobic conditions in ‘Anaerobic’. FPA levels below the detection limit are shown as zero. The experiment is the same as in Fig. 2. Symbols: (*open* *circle*) CDW ‘Aerobic’, (*filled circle*) CDW ‘Anaerobic’, (*open* *square*) FPA ‘Aerobic’ and (*filled* *square*) FPA ‘Anaerobic’

To investigate the physiological response of *C. uda* to a shift from aerobic to anaerobic growth conditions, two shake-flask cultures were initially grown under aerobic conditions. These cultures immediately grew and produced FPA at a slightly faster rate than the aerobic cultures described in the previous experiment (cf. Figs. 1, 3). After 36-h incubation, the paper stopper of one of the aerobically grown cultures was exchanged with a butyl rubber stopper and the gas phase was replaced by N_2_. The other culture was retained under aerobic conditions. The culture shifted to anaerobic conditions ceased producing FPA, and cell growth was significantly reduced (Fig. 3). The shift to anaerobic conditions also stopped production of EGA and extra-cellular protein (results not shown) and fermentation products were immediately produced (Fig. 2b), dropping the pH to 6.0. In contrast, almost no fermentation products were detected in the aerobically grown culture (Fig. 2a), and the pH only declined to ~6.9 after 240 h. The primary fermentation products were ethanol, formic acid and acetic acid—formic acid being the dominant product. At the end of the incubation, the total organic C content of the fermentation products corresponded to ~42 % of the initial C content in α-cellulose (20 g l^−1^).

### Growth and cellulase production under anaerobic conditions

**Fig. 4:**
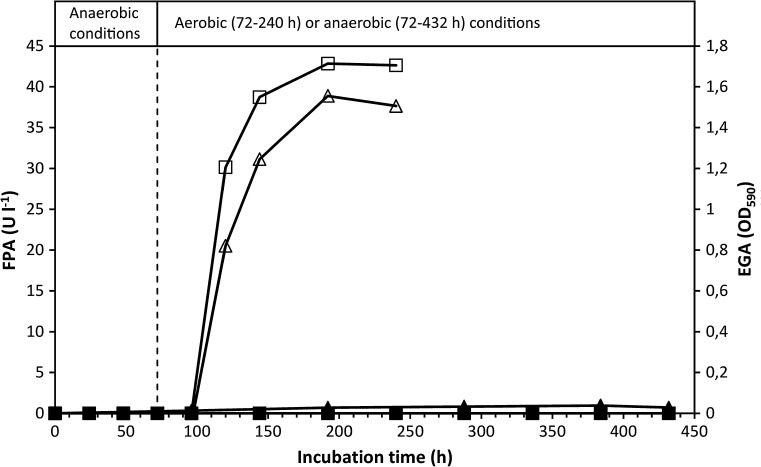
FPA and EGA levels in cultures grown on α-cellulose (20 g l^−1^) under anaerobic conditions (labelled ‘Anaerobic’) or under initially anaerobic conditions followed by aerobic conditions (labelled ‘Aerobic’). The *broken vertical line* indicates the switch to aerobic conditions in ‘Aerobic’ at 72 h. FPA levels below the detection limit are shown as zero. Symbols: (*open* *square*) FPA ‘Aerobic’, (*filled square*) FPA ‘Anaerobic’, (*open* *triangle*) EGA ‘Aerobic’ and (*filled triangle*) EGA ‘Anaerobic’

To further explore the apparent absence of cellulase production by anaerobically cultivated *C. uda* (cf. Fig. 3), anaerobic cultures were prepared and maintained for 432 h (hereafter referred to as ‘Anaerobic’), or shifted to aerobic conditions after 96-h incubation (referred to as ‘Aerobic’). The FPA and EGA of ‘Aerobic’ sharply increased after the switch to aerobic conditions (Fig. 4). The EGA reached a maximal level, which corresponds to a CMCase activity of approximately 2600 U l^−1^. In ‘Anaerobic’, the FPA remained below the detection limit throughout the incubation period, whereas the EGA marginally increased but never exceeded 3 % of the highest EGA activity measured in ‘Aerobic’. Based on the ammonium assimilation, ‘Anaerobic’ produced approximately 0.6 g CDW l^−1^, versus 6.2 g CDW l^−1^ in ‘Aerobic’. ‘Anaerobic’ exhibited a low but steady accumulation of fermentation products, paralleling a continuous pH decline (from 7.4 to 6.5 after 432 h; results not shown). After 432 h, the organic C content of the fermentation products in ‘Anaerobic’ corresponded to ~35 % of the supplied α-cellulose C.

### Induction of cellulase activity by cellulose in dense anaerobic culture

**Fig. 5:**
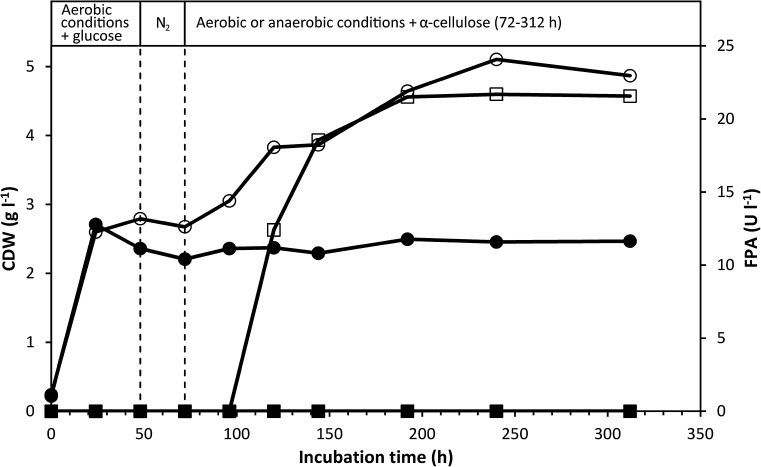
CDW and FPA levels in *C. uda* cultures initially grown aerobically on glucose as the initial substrate (5 g l^−1^). After 48 h (*first vertical dotted line*), the gas phase of all cultures was replaced with N_2_. After 72 h (*second vertical dotted line*), α-cellulose (10 g l^−1^) was added to all cultures and the gas phase of ‘Aerobic’ was reverted to atmospheric air, while ‘Anaerobic’ remained anaerobic. FPA levels below the detection limit are shown as zero. Symbols: (*open* *circle*) CDW ‘Aerobic’, (*filled* *circle*) CDW ‘Anaerobic’, (*open* *square*) FPA ‘Aerobic’ and (*filled* *square*) FPA ‘Anaerobic’

In the previous experiment, the lack of cellulase production by anaerobically cultivated *C. uda* (Fig. 4) might have resulted from the low concentration of active cells, which could reduce the cellulase activity to near the detection limit. To investigate this, we incubated *C. uda* cells in a medium initially containing glucose to induce rapid aerobic upgrowth. Glucose was chosen as the initial growth substrate to produce high cell densities and reduce initial cellulase activity prior to induction of cellulase production. After 48-h aerobic growth on glucose, the gas phase in all flasks was replaced with N_2_, and after additional 24-h incubation, sterile α-cellulose (10 g l^−1^) was added to all flasks. Immediately after cellulose addition, two of the flasks were shifted to aerobic growth conditions (referred to as ‘Aerobic’) by replacing the butyl stopper with paper stoppers. The remaining two flasks were maintained under anaerobic conditions (referred to as ‘Anaerobic’) for 240 h (Fig. 5).

**Fig. 6:**
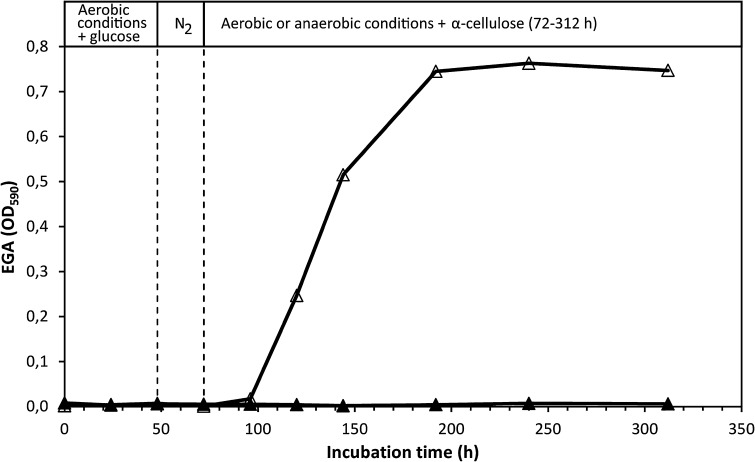
EGA levels in *C. uda* cultures initially grown aerobically on glucose as the initial substrate (5 g l^−1^). After 48 h (*first vertical dotted line*), the gas phase of all cultures was replaced with N_2_. After 72 h (second vertical dotted line), α–cellulose (10 g l^−1^) was added to all cultures and the gas phase of ‘Aerobic’ was reverted to atmospheric air, while ‘Anaerobic’ remained anaerobic. Same experiment as in Fig. 5. Symbols: (*open* *triangle*) EGA ‘Aerobic’ and (*filled* *triangle*) EGA ‘Anaerobic’

Glucose was instantly consumed during the initial phase of aerobic growth and was exhausted after 24 h (results not shown). The rapid glucose consumption was paralleled by cell growth, which ceased when the glucose was depleted from the medium. At 48 h, a cell density of 2.4–2.8 g CDW l^−1^ was calculated from analyses of C and N of washed culture pellets. In ‘Aerobic’, α-cellulose addition after 72-h incubation restored ammonium consumption and cell growth, and stimulated sharp increases in EGA (Fig. 6) and FPA (Fig. 5). In contrast, α-cellulose addition did not stimulate FPA and EGA production in ‘Anaerobic’, and only very little cell growth and ammonium assimilation occurred. Fermentation products accumulated more slowly (results not shown) than in the previously described experiments (Fig. 2b, and experiment shown in Fig. 4). No fermentation products appeared in ‘Aerobic’. At 312 h, fermentation products produced from cellulose amounted to ~19 % of added cellulose C. Throughout the 312-h incubation period, the FPA of ‘Anaerobic’ never exceeded the detection limit (10 U l^−1^; see Fig. 5), and the EGA production remained very low (<1 % of the highest activity obtained in ‘Aerobic’; see Fig. 6).

### Anaerobic growth on cellobiose and glucose

To confirm that *C. uda* can grow anaerobically on the two primary products of cellulose hydrolysis (cellobiose and glucose), growth kinetics on these substrates (10 g l^−1^) under anaerobic conditions were investigated. Instantaneous anaerobic growth was observed on both cellobiose and glucose; the C/N analyses yielded growth amounting to 1.3 and 1.1 g CDW l^−1^, respectively. The respective growth yields on cellobiose and glucose were 0.15 and 0.10 g CDW g^−1^ substrate consumed. At the end of the incubation (192 h), the fermentation products and cell biomass accounted for 85.4 and 16.9 %, respectively, of the consumed cellobiose-C, and 81.7 and 12.6 %, respectively, of the consumed glucose-C. No EGA was produced on either substrate. Fermentation products appeared in the growth medium from the start of the incubation and accumulated throughout the incubation period, while the pH decreased from 7.4 to 6.5. The results are summarised in Table 1.

**Table 1:** Anaerobic growth of *C. uda* on glucose and cellobiose

Parameters	Substrate	
	Glucose (10 g l^−1^)	Cellobiose (10 g l^−1^)
OD_600_ stationary phase	2.83–2.95	3.48–3.79
Ammonium consumption (mmol l^−1^)	5.6	6.4
Growth efficiency (g CDW g^−1^ substrate consumed)	0.10	0.15
Distribution of substrate C metabolized in (%):		
Fermentation products	81.7	85.4
Cells	12.6	16.9
pH at end of incubation (192 h)	6.5	6.5

### Is EGA bound to cellulose and cells during anaerobic growth?

To investigate whether *C. uda* produces cell-bound cellulases under anaerobic conditions (which would not be detected in the standard EGA assay using culture supernatant), we performed a modified and more sensitive EGA assay on whole culture broth (containing cells and α-cellulose) and the culture supernatant. The modified assay would also detect cellulase activity bound to cellulose (see ‘Discussion’ section). To ensure high cell numbers in the assay incubations (i.e. high sensitivity), the volume of culture broth (and supernatant) was raised from 20 µl in the standard EGA assay (cf. Materials and methods section) to 500 µl. In the modified assay, 500 μl of culture broth/supernatant was added to 500 µl of phosphate buffer (0.1 mol l^−1^, pH 7.0). *C. uda* anaerobically cultured on α-cellulose (10 g l^−1^) was sampled at 0, 72, 168 and 360 h and subjected to both modified and standard EGA assays. In the modified assay, the EGA values were 6–18 % higher in whole culture broth than in culture supernatants of the same sample, indicating that some cellulase activity was bound to cellulose and/or cells. The results of the standard EGA assay were consistent with those of our previous anaerobic growth experiments (e.g. Figure 4); that is, the EGA in the culture supernatant very slowly increased throughout the incubation period, but remained very low at all times. These data (and others not shown) could indicate that very low amounts of cellulase activity are produced constitutively under anaerobic conditions (see ‘Discussion’ section).

### Additional tests of *C. uda* strains and growth media

We found no evidence for significant *de novo* synthesis of either cell-bound or cell-free FPA/EGA activity by *C. uda* anaerobically grown on α-cellulose (Figs. 3, 4, 5, 6) as the sole carbon and energy source, which is at variance to results obtained in a few previous studies (Dermoun and Belaich 1988; Reguera and Leschine 2001)—see ‘Discussion’ section. If our laboratory strain of *C. uda* (DSM 20108) had spontaneously mutated, it would no longer represent the type strain used in many previous studies. To test whether such mutation was responsible for this discrepancy, we repurchased *C. uda* (DSM 20108) from DSMZ and compared its anaerobic cellulase production in medium containing α-cellulose with that of *Cellulomonas sp.* (ATTC 21399) obtained from ATCC (Mannasas, Virginia, USA). These strains should be identical (ATCC; pers. comm.) and recognised as *C. uda* (Stackebrandt and Kandler 1979). Thorough testing showed that these newly purchased strains behaved identically to our original strain when anaerobically cultivated in media containing α-cellulose.

Medium composition may greatly influence production levels of microbial metabolites such as extra-cellular enzymes. Accordingly, anaerobic batch cultivations were also conducted in the exact medium used by Dermoun and Belaich (1988) for anaerobic cultivation of *C. uda* with different cellulose substrates in a bioreactor at pH 7. We tested this medium supplemented with MOPS (42 g l^−1^) for better pH control or with pH control to the initial value of 7.0 by daily additions of sterile NaOH. Neither of these modified growth media resulted in significant anaerobic production of cellulase activity when incubated with α-cellulose as carbon and energy substrate.

Finally, in order to rule out the unlikely scenario that *C. uda* would perform differently when cultivated on another crystalline cellulose substrate, we performed growth experiments with Avicel (a frequently used inducer of cellulase activity) as sole energy and carbon source in aerobic and anaerobic cultures. In Avicel-grown cultures, FPA and AVA in anaerobic incubations were below or at the detection limit and EGA maximally amounted to 3.0 % of that measured in aerobic cultures—as observed for α-cellulose-grown cultures.

## Discussion

Our results confirmed that *C. uda* readily grows on cellulose aerobically, as reported in several previous investigations (Deconinckchosson 1988; Dermoun and Belaich 1985; Haggett et al. 1979). After 240 h of aerobic cultivation in media containing 10 and 20 g l^−1^ α-cellulose, the growth yield (~0.42 g CDW g^−1^ substrate consumed) and degree of α-cellulose hydrolysis (77–81 %) were similar to those reported by Dermoun and Belaich (1988), who aerobically cultivated *C. uda* on other purified cellulose products (CC41, Avicel, MN300). Metabolic activity (e.g. ammonium assimilation) of aerobic cultures incubated with α-cellulose for 240 h was greatly stimulated by glucose addition, indicating that *C. uda* growth was slowed by substrate availability towards the end of the incubation period (Fig. 1), and became controlled by the rate of glucose equivalents released from the remaining α-cellulose, since FPA was at its maximum level at this time.

A shift from aerobic to anaerobic conditions during the initial phase of rapid growth immediately halted the production of FPA (Fig. 3) (and of EGA; results not shown). To further investigate this finding, cultures were incubated under anaerobic conditions throughout the incubation period. The very sensitive EGA assay showed that cellulase activity marginally increased—but remained very low—during the extended (432 h) incubation period, while the FPA never exceeded the detection limit (Fig. 4). The very low increase in EGA might be interpreted as constitutive cellulase synthesis since cell growth during this period was negligible as based on ammonium assimilation (consumed NH_4_^+^ after 432 h: ~4.5 mM). Another explanation could be that the cellulase enzymes (added with the inoculum) slowly desorbed from the cellulose substrate during the prolonged incubation period. Furthermore, small amounts of oxygen might have entered the culture medium (which contained no oxygen-scavenging reducing agents) by diffusion and/or sampling, which may have induced the production of very small amounts of cellulase. Other experimental attempts to magnify any putative anaerobic cellulase production similarly yielded no FPA or EGA. In these experiments, cellulose was added to dense and starving anaerobic *C. uda* cultures with low initial cellulase activity (Figs. 5 and 6). Therefore, according to the cellulase activity assays in the present study and the levels of aerobic cellulase production observed, it appears that cellulase production is not induced by *C. uda* in the absence of oxygen. A very low constitutive production of cellulase activity in anaerobically cultivated *C. uda* cannot be completely ruled out; however, as the EGA production never exceeded 3.0 % of the aerobic EGA production, *C. uda* is not an anaerobic cellulose degrader sensu stricto when compared to strictly anaerobic cellulase producers such as *Clostridium* spp. The FPA assay is insufficiently sensitive to detect the small increases in hydrolytic activities in anaerobic cultures.

Relatively high amounts of fermentation products (>18 % of the added substrate, based on the organic C content) accumulated in cultures with even very low cellulase activity (Figs. 4, 5) as compared to aerobic activities. This shows that the cellulase system of *C. uda* is efficient and stable over prolonged times (see the FPA levels of ‘Anaerobic’ in Fig. 3). According to our calculations, FPA levels far below the detection limit of our assay (i.e. 10 U l^−1^) can easily account for the cellulose hydrolysis necessary to produce the amount of organic C present in fermentation products and cell biomass at the end of anaerobic incubations (Figs. 4, 5). These calculations assume similar catalytic hydrolysis rate on α-cellulose as determined for filter paper. It should be noted that *C. uda* may ferment the storage compounds trehalose and glycogen (Schimz and Overhoff 1987), which might also contribute to fermentation products. This possibility was not further addressed in this study.

The putative absence of anaerobic cellulase production most likely is not due to the decrease in pH caused by the fermentation products accumulating during anaerobic incubation. Prior to imposing anaerobic growth conditions, the pH was high (>7.2) in all *C. uda* cultures and remained high throughout the incubation period also in the dense anaerobic culture supplemented with cellulose (Fig. 5). Repression of cellulase production by glucose and/or cellobiose is not a likely scenario either, since these compounds did not accumulate in anaerobic or aerobic cultures. In the experiment depicted in Figs. 5, 6, HPLC analysis showed that the initially added glucose was not detectable (detection level of 0.1 mmol l^−1^) after 24 h and no glucose or cellobiose was detected in samples from the remainder of the 312-h incubation period.

Many studies have described binding of cellulases to their insoluble substrates (Beguin et al. 1977; Coutinho et al. 1992; Sandercock et al. 1996), and the ratio of free versus substrate-bound cellulase activity may change with the age of the culture (Beguin et al. 1977; Hagerdal et al. 1978; Vladuttalor et al. 1986). Cell-bound cellulases have been indicated in *C. uda* (Stoppok et al. 1982) and other *Cellulomonas* species (Lamed et al. 1987; Rodriguez and Volfoa 1984). However, Christopherson et al. (2013) analysed the whole genomes of three *Cellulomonas* species (not including *C. uda*) and found no indications of cell-bound cellulases. Assuming that *C. uda* indeed produces cellulases under anaerobic conditions, these might be exclusively cell bound as observed for typical anaerobic cellulolytic microorganisms and/or bound to the cellulose substrate as described above. Cell-bound or cellulose-bound cellulases would not be detected by our standard EGA assay protocol, which examines cell-free supernatants. This underestimation of cellulase activity is especially pronounced in culture broths containing low cellulase and high cellulose concentrations (unpublished results on *C. uda*). To survey whether *C. uda* produces cell-bound or substrate-bound cellulase activity under anaerobic conditions, we performed a modified and more sensitive EGA assay that compared the activities in whole culture broth (containing cells and α-cellulose) and cell-free culture supernatant. The sensitive assay using a dyed artificial substrate revealed that the EGAs were indeed lower in the culture supernatant (by 6–18 %) than in whole culture broth of the same sample (collected after 0, 72, 168 and 360 h of anaerobic incubation). These results suggest the existence of cell-bound and/or cellulose-bound activity, albeit at low levels. The observed differences between supernatant and whole culture broth were not detectable in the standard EGA assay, in which the assayed volume is 25 times smaller than in our modified method. EGA activities in anaerobic cultures never exceeded 3.0 % of those determined in aerobic cultures.

Published data on cellulase activities produced by *C. uda* during anaerobic growth are very scarce. Reguera and Leschine (2001) reported an average CMCase activity in anaerobic cultures of 1626 U/mg protein compared to 52 U/mg protein in aerobic cultures, but without reporting the corresponding protein concentrations. In the study of Dermoun and Belaich (1988) it is stated: ‘Synthesis of the enzymatic machinery was not found to be dependent on the growth atmosphere’ although their data (cf. Table 1, pg. 401) indicate production of lower CMCase levels under anaerobic conditions—when converting the reported values of cell growth yields to cellular protein. It is generally accepted that comparison of published cellulase activities is usually very difficult, due to differences in assay conditions and/or because enzyme activities are stated in units which cannot be easily interconverted. Nonetheless, in the present study levels of total cellulase activity (FPA or AVA) were always below the detection limit in cultures grown anaerobically and endocellulase activities (EGA) were <3 % of the maximum values measured in parallel aerobic cultures (Figs. 4 and 6). This was in good agreement with highly different levels of supernatant protein measured in aerobic (375 mg/L) and anaerobic (17 mg/L) cultures after 6 days of growth (data relating to Fig. 4), which in turn correlated with the low cell yields observed in anaerobic cultures.

In summary, our results show that *C. uda* grows rapidly and excretes free cellulases during aerobic cultivation on α-cellulose or Avicel as sole carbon and energy source. Notably, no substantial *de novo* synthesis of cellulase activity (either cell-bound or cell-free) was observed under anaerobic growth conditions. However, the cells demonstrated significant fermentative activity (and limited growth), metabolising the hydrolysis products released from cellulose. These hydrolysis products were likely derived either from cellulase activity present in the 5 % (v/v) inoculum and/or resulted from extremely low constitutive production of cellulases. From an application perspective, our results demonstrate that *C. uda* is not a prospective cellulase producer under anaerobic conditions, although it metabolically behaves as a true facultative anaerobe on soluble carbon substrates (Marschoun et al. 1987; Dermoun and Belaich 1988). Further studies are required to elucidate why *C. uda* only produces negligible cellulase activity (FPA, AVA and EGA) under anaerobic conditions, and whether this deficiency extends to other published cellulolytic species of *Cellulomonas*.
